# Determinants of viral load non-suppression among people living with HIV on anti-retroviral therapy in Kumasi, Ghana

**DOI:** 10.4314/gmj.v55i2.3

**Published:** 2021-06

**Authors:** David KO Ansah, Emmanuel Kumah, Vitalis Bawontuo, Peter Agyei-Baffour, Emmanuel K Afriyie

**Affiliations:** 1 Faculty of Health and Allied Sciences, Catholic University College of Ghana, P.O. Box 363, Fiapre –Sunyani; 2 Policy, Planning, Monitoring & Evaluation Unit, Komfo Anokye Teaching Hospital, P.O. Box 1934, Kumasi, Ghana; 3 Department of Health Policy, Management and Economics, School of Public Health, Kwame Nkrumah University of Science and Technology, Kumasi, Ghana; 4 Laboratory Services Directorate, Komfo Anokye Teaching Hospital, P.O. Box 1934, Kumasi, Ghana

**Keywords:** HIV/AIDS, anti-retroviral therapy, viral load suppression, virological failure, Ghana

## Abstract

**Objectives:**

To determine the rate and factors associated with viral load non-suppression among adults living with HIV/AIDS on active anti-retroviral therapy (ART).

**Design:**

A retrospective cross-sectional study

**Setting:**

Three ART clinics in Kumasi, Ghana

**Participants:**

All HIV-infected adults who were ≥18 years and on active ART for 12 months and whose viral load had been estimated were included.

**Main outcome measure:**

Unsuppressed viral load among patients on ART

**Results:**

In all, 483 HIV patients were included in the study, with 369 (76.4%) achieving viral load suppression. Gender, educational level, comorbidity status, and duration on ART were independently associated with viral non-suppression (p < 0.05).

**Conclusions:**

This study has revealed that the rate of viral suppression in the study area is lower than the UNAIDS 90% target. The findings have implications on designing new and stemming up implementation of existing interventions to improve the rate of viral suppression among patients in the study area. It is also necessary that more of such studies are replicated in other parts of the country to identify risk factors for virological failure among patients on ART.

**Funding:**

No funding was obtained for this study

## Introduction

HIV/AIDS is a leading cause of global disease burden, with over 39 million deaths and more than 36 million people currently living with the condition.^1^ Despite great advancements in anti-retroviral therapy (ART) and worldwide progress towards treatment and prevention programs, about 2 million people get infected with HIV every year. Sub-Saharan Africa has the highest burden of the global HIV epidemic, recording over 75% deaths and 65% new infections in 2017.^2^

The global community has made a repeated call for the end of the HIV epidemic. In 2014, the Joint United Nations Program on HIV/AIDS (UNAIDS) launched the 90-90-90 targets to diagnose 90% of all HIV-positive persons, providing ART for 90% of those diagnosed and achieving viral suppression for 90% of those treated by 2020. A 95-95-95 goal had also been set for 2030.^3^ In 2017, the global HIV care continuum stood at 75-79-81.^3^

Available evidence indicates that early placement of patients on ART and achievement of viral load suppression reduces mortality and HIV transmission and improves the quality of life.^4^ Viral load monitoring increases life expectancy^5^ and ensures a reduction in misdiagnosis of treatment failure, leading to effective utilization of limited resources.^6^ However, while access to ART has been scaled up worldwide, virological failure remains a common problem for HIV patients.

For instance, in 2014/2015, only 32% of the global figure of 36.9 million HIV-positive people achieved viral load suppression. Also, a study conducted by Levi et al. in 69 countries found that viral load suppression was between 68% (in Switzerland) and 7% (in China). The lowest achievement rates were found in low and middle-income countries.^7^

Ghana, like many countries, is striving towards attaining the 90-90-90 UNAIDS targets. In 2016, the government adopted the World Health Organization (WHO) policy of “treat all”, which is providing ART to all people living with HIV/AIDS (PLWH) irrespective of their CD4 count – a mechanism used previously as the cutoff to start treatment. Although active ART is a major component of the country's range of comprehensive care, the Ghana AIDS Commission (GAC) puts the current rate of virological failure around 34% (https://www.ghanaids.gov.gh), an indication that the country is far from attaining the 90-90-90 targets.

Studies have shown that various factors are associated with viral load non-suppression.^8^, ^9^. Identifying and managing these factors is vital to achieving a high treatment success rate and improving the quality of life of people living with HIV/AIDS.^9^ However, limited evidence exists on the determinants of viral load non-suppression among people living with HIV/AIDS on active ART in Ghana. A 2017 longitudinal study by Owusu et al. to determine the prevalence and risk factors of virological resistance among HIV-1-positive children on ART at Komfo Anokye Teaching Hospital, Kumasi, Ghana, found that subjects whose parents were unemployed had 5.4 (1.4-20.9) chances of virological failure compared to those with employed parents.^10^ Recently, Lokpo and colleagues conducted a retrospective study to assess the rate and associated factors of viral suppression in the Ho Municipality of the Volta Region of Ghana. The authors found regular ART clinic attendance and treatment >3 years as factors associated with viral suppression.^11^

The present study builds on the above studies by determining the rate and factors associated with viral load non-suppression among adults living with HIV/AIDS on active ART at Kumasi in the Ashanti Region of Ghana. Findings could inform policy decisions and strategies in improving care and attaining the 90-90-90 and the 95-95-95 targets.

## Methods

### Study design and setting

We conducted a retrospective cross-sectional study from January 2018 to December 2019 at three ART clinics in Kumasi, Ashanti Region. The clinics were Manhyia Government Hospital ART Clinic, Suntreso Government Hospital ART Clinic and Kumasi South Hospital ART Clinic. The Manhyia Government Hospital ART Clinic, which became operational in June 2016, was the last among the three operationalized. Ashanti Region is the most populous region in Ghana and accounts for an estimated 52,760 (18.5%) out of 284, 860 adults (15 years and over) living with HIV and AIDS in the country.^12^ The three ART clinics had 4,060 HIV-infected adult clients actively enrolled in ART as of December 2019: 660 at Manhyia Hospital ART Clinic, 1,500 at Suntreso Government Hospital ART Clinic, and 1,900 Kumasi South Hospital ART Clinic.

### Study population and sample

We included all HIV-infected adults who were 18 years and above and on active ART for 12 months and whose viral load had been estimated. All HIV-infected persons who were above 18 years of age and had been on ART for 12 months and over but did not have their viral load results readily available were excluded from the study. All those who had been lost to follow up or dead were also excluded from the study.

### Study variables

The primary outcome variable under study was unsuppressed viral load among patients on ART. In line with WHO categorization for low and middle-income countries (LMICs), patients with viral load of > 1000 copies of viral ribonucleic acid (RNA) per ml were considered as virally non-suppressed, whereas those with a viral load of < 1000 RNA copies per ml were taken as virally suppressed.^6^ Eleven explanatory variables classified into socio-demographic factors and patient clinical characteristics were included in the analysis. The socio-demographic variables were age, sex, educational level, marital status, occupation, and religion. The patient clinical characteristics included comorbidity, weight, blood pressure, HIV type and duration on ART (years).

### Data Collection Tool and Procedure

Socio-demographic characteristic and clinical information on the patients were extracted from registers and patient folders from January 2018 to December 2019. A designed data collection tool reflecting the various variables under study was used to gather the data. Three health care (data) professionals at each facility were trained and supported to collect and compile the data. All the data gathered were audited and verified to check for completeness and quality.

### Data Analysis

All analyses were done using Statistical Package for Social Sciences (SPSS Inc., Chicago, USA (http://www.spss.com) version 20. Descriptive statistics: mean, frequencies, and percentages were used to describe the patients' demographic and socioeconomic related characteristics. We estimated the proportion of virological failure by dividing the total number of patients with no viral suppression by the total number of patients included in the study. We dichotomized the outcome variable (1 = viral load non-suppressed and 0 = viral load suppressed). Bivariate analysis was then performed for all of the independent variables with the outcome variable. Using variables with a p-value of up to 0.05 from the bivariate analysis, a multinomial logistic regression analysis was carried out to determine the independent determinants of viral non-suppression. Variables with significant associations with virological failure were identified based on the odds ratio (OR) with a 95% confidence interval (CS) and p-values ≤ 0.05.

### Ethical Considerations

Ethical clearance was sought from Ghana Health Service's Ethical Review Committee of the Research and Development Division (No. GHS-ERC-048/10/19). In addition, approval was obtained from the Ashanti Regional Health Directorate (ARHD), the Kumasi Metro Health Directorate (KMHD), and the Management of the selected facilities and the anti-retroviral therapy (ART) clinics. Data retrieved were not linked to any patient.

## Results

### Patient characteristics

Socio-demographic and clinical characteristics of the study participants are shown in Table 1. The study participants consisted of 483 patients. The majority of them were female (73.1%), below the age of 40 years (41.2%), married (49.3%), employed (83.9), and having a basic level of education (52%) (Table 1).

**Table 1:** Demographic characteristics of patients included in the study (n = 483)

Variable	Frequency (%)
**Gender:**	
**Male**	130 (26.9)
**Female**	353 (73.1)
**Age:**	
**< 40**	199 (41.2)
**40-49**	149 (30.8)
**≥ 50**	135 (27.9)
**Marital Status:**	
**Single**	158 (32.7)
**Married**	238 (49.3)
**Other (cohabiting, widowed, divorced)**	81 (16.8)
**Educational level:**	
**None**	34 (7)
**Basic**	251 (52)
**Secondary**	169(35)
**Tertiary**	29 (6)
**Employment Status:**	
**Employed**	405 (83.9)
**Unemployed**	78 (16.1)
**Religion:**	
**Christian**	358 (74.1)
**Muslim**	112 (23.2)
**Traditionalist**	13 (2.7)

Most of the patients (66.7%) had comorbidities such as TB (29), hypertension (155), asthma (22) and diabetes mellitus (116). More than half (59.4%) had blood pressure above 140/90. Also, the majority of the participants were HIV-1 patients (84.1%), with bodyweight below 70kg (51.2%), and had been on ART for less than five years (62.1%) (Table 2).

**Table 2:** Clinical Characteristics of patients included in the study (n = 483)

Variable	Frequency (%)
**Comorbidity status:**	
**Comorbidity**	322 (66.7)
**No Comorbidity**	161 (33.3)
**Blood Pressure:**	
**< 130/80**	195 (40.6)
**130-139/80-89**	156 (32.1)
**≥140/90**	132 (27.3)
**Type of HIV Infection:**	
**HIV-1**	406 (84.1)
**HIV-2**	5 (1)
**HIV-1 & 2**	72 (14.9)
**Weight:**	
**< 70kg**	250 (51.8)
**≥ 70kg**	233 (48.25)
**Duration on ART:**	
**< 5 years**	300 (62.1)
**≥ 5 years**	183 (37.9)

### The proportion of viral non-suppression

Based on the definition of virological failure we adopted for this study (i.e. viral load of >1000 RNA copies/ml), 114 (23.6%) out of the 483 patients were identified as having no viral suppression. The remaining 369 (76.4%) patients had their viral loads count as either target not detected (TND) (n = 248, representing 51.3%) or <1000 RNA copies/ml (n = 121, representing 25.1%).

### Factors associated with viral non-suppression

Our bivariate analysis (Table 3) showed that employment status, religion and type of HIV infection had no significant association with viral non-suppression. However, eight variables, namely: gender, age, marital status, educational level, comorbidity status, blood pressure, body weight, and duration on ART were significantly associated with viral non-suppression.

**Table 3:** Bivariate test analysis of factors associated with viral non-suppression

Variable	Virally Suppressed	Viral Non-Suppressed	p value
**Gender:**			
**Male**	108 (83.1%)	22 (16.9%)	
**Female**	261 (73.9%)	92 (26.1%)	0.021
**Age:**			
**< 40**	150 (75.4%)	49 (24.6%)	
**40-49**	111 (74.5%)	38 (25.5%)	0.023
**≥ 50**	108 (80%)	27 (20%)	
**Marital Status:**			
**Single**	123 (77.8%)	35 (22.2%)	
**Married**	180 (75.6%)	58 (24.4%)	0.024
**Other (cohabiting, widowed, divorced)**	62 (76.5%)	19 (23.5)	
**Educational level:**			
**None**	25 (73.5%)	9 (26.5%)	
**Basic**	194 (77.3%)	57 (22.7%)	
**Secondary**	128 (75.7%)	41 (24.3%)	0.001
**Tertiary**	20 (69%)	7 (31%)	
**Employment Status:**			
**Employed**	311 (76.8%)	94 (23.2%)	
**Unemployed**	58 (74.4%)	20 (25.6%)	0.514
**Religion:**			
**Christian**	276 (77.1%)	82 (22.9%)	
**Muslim**	86 (76.8%)	26 (23.2%)	0.337
**Traditionalist**	9 (69.2%)	4 (30.8)	
**Comorbidity status:**			
**Comorbidity**	252 (78.3%)	70 (21.7%)	
**No Comorbidity**	117 (72.7%)	44 (27.3)	0.016
**Blood Pressure:**			
**< 130/80**	146 (74.9%)	49 (25.1%)	
**130-139/80-89**	122 (78.1)	34 (21.9)	
**≥ 140/90**	102 (77.4)	30 (22.6%)	0.032
**Type of HIV Infection:**			
**HIV-1**	312 (76.8%)	94 (23.2)	
**HIV-2**	4 (80%)	1 (20%)	0.617
**HIV-1 & 2**	55 (76.4%)	17 23.6%)	
**Weight:**			
**< 70**	185 (74%)	65 (26%)	
**≥ 70**	184 (78.9%)	49 (21.1%)	0.002
**Duration on ART:**			
**< 5 years**	230 76.7%)	70 (23.3%)	
**≥ 5 years**	139 (75.9%)	44 (24.1)	0.0412

Using a multinomial logistic regression analysis, we observed that the odds of having virological failure were independently associated with gender, educational level, comorbidity status, and duration on ART (Table 4). For instance, we observed that the likelihood of developing virological failure for male patients was 1.3 times (OR = 1.28, 95% CI: 1.19-1.38) higher compared with female patients. Similarly, patients who had attained basic (OR = 0.62, CI: 0.14-2.84), secondary (0.6 OR = 0.57, CI: 0.13-2.17) and tertiary (OR = 0.53, CI: 0.12-2.11) levels of education were less likely, compared with patients with no level of education, to develop virological failure.

**Table 4:** Multinomial logistic regression analysis of factors associated with viral non-suppression among adult HIV patients on ART

Variable	OR	95% CI	p-value
**Gender:**			
**Female**	Ref.		
**Male**	1.28	1.19 – 1.38	0.003
**Age:**			
**< 40**	Ref.		
**40-49**	1.13	0.59 – 1.95	0.85
**≥ 50**	1.64	0.97 – 3.29	0.301
**Marital Status:**			
**Single**	Ref.		
**Married**	1.12	0.65 – 1.90	0.683
**Other (cohabiting, widowed, divorced)**	0.62	0.27 – 1.61	0.473
**Educational level:**			
**None**	Ref.		
**Basic**	0.62	0.14 – 2.84	0.021
**Secondary**	0.57	0.13 – 2.17	0.001
**Tertiary**	0.53	0.12 – 2.11	0.002
**Comorbidity status:**			
**No Comorbidity**	Ref.		
**Comorbidity**	2.42	2.33 – 2.51	0.012
**Blood Pressure:**			
**< 130/80**	Ref.		
**130-139/80-89**			
**≥ 140/90**	0.98	0.63 – 0.96	0.951
**Weight:**			
**< 70**	Ref.		
**≥ 70**	1.19	1.02 – 1.39	0.777
**Duration on ART:**			
**< 5 years**	Ref.		
**≥ 5 years**	1.18	1.10 – 1.26	0.042

Further, compared with patients with no comorbidity, patients having comorbidities were 2.4 times (OR = 2.42, CI: 2.33-2.51) more likely to develop virological failure. Finally, patients on ART for 5 years and above were 1.2 times (OR = 1.18, CI: 1.10-1.26) more likely than patients who had been on ART for less than 5 years to develop virological failure.

## Discussion

We conducted this study to determine the rate and factors associated with viral load non-suppression among adults living with HIV/AIDS on active ART in the Kumasi Metropolis of the Ashanti Region of Ghana. We found that 23.6% of patients in care on ART with a viral load on record were virally unsuppressed. Our multinomial logistic regression model revealed that gender, educational level, comorbidity status, and duration on ART were independently associated with viral non-suppression.

Although the viral suppression rate (76.4%) in this study is lower than the UNAIDS 90% target for viral suppression on ART, the proportion of non-suppression (23.6%) is an improvement compared to the national rate of 34%. A study conducted in Ho in the Volta Region of Ghana reported a virological failure of 31.3% (i.e. 89 non-suppression out of 284 HIV-positive patients).^11^ What this implies is that there might be variations in the proportion of HIV-infected people achieving viral load suppression in different parts of the country.

Our analysis revealed that the likelihood of not achieving viral load suppression among male patients was 1.3 times more likely than that of female patients. This finding is consistent with studies conducted in other African countries, including Burkina Faso,^13^ Ethiopia,^14^ Nigeria^15^ and Swaziland.^16^ The reason females were less prone to virological failure might be their high health-seeking behaviour, as reported in the literature.^17^,^18^ It has been noted that health policies and the effects of female empowerment within healthcare systems strengthen women's access to health services.^13^,^15^,^18^ Also, gender asymmetry in PLWH's health-seeking behaviour has been attributed to the representations of masculinity which are fully implicated in the cultural construction of males' reluctance to attend care for HIV patients.^18^ According to Bila & Egrot,^18^, the values associated with this masculinity cause males to run greater health, economic and social risks. Thus, there is a need for effective strategies, such as health education, sensitization and campaigns, to enhance health-seeking behaviour among male HIV-infected patients.

Our bivariate analysis demonstrated that age was significantly associated with virological failure. However, in the multinomial regression model, age was not an independent predictor of virological failure. Studies from Kenya do not support this observation,^19^ Cameroon^20^ and Mozambique^21^, which reported a greater risk of virological failure among younger patients.

We observed that as the educational level of the study participants decreased, the risk of developing virological failure increased. This concurs with the literature that people with HIV who have lower educational attainment have poorer treatment outcomes when placed on ART.^22^,^23^ To reverse this trend, there is a need for targeted education and the provision of relevant information on ART to support these HIV patients with lower levels of education.

We found that most patients (about 78%) had comorbidities, and comorbidity status was significantly associated with viral load non-suppression. This is consistent with a study conducted in Uganda, which found that HIV patients with active TB were more likely to be virologically non-suppressed. However, a recent study conducted in Western Cape, South Africa, found no association between detectable HIV viral load and risk of developing comorbidities.^24^ The association between comorbidity and virological failure found in this study could be explained by the likely effect of the patients suffering from two or more chronic conditions, with associated stigma and discrimination.

Duration on ART was significantly associated with viral load non-suppression, and patients on ART for more than 5 years had increased odds of developing virological failure. This finding is supported by other studies conducted in Gabon^25^ and Mozambique.^26^ The possible explanation to this outcome might be that as the duration on ART increased, the chances of developing poor adherence and drug side effects also increased.^27^ For instance, in a study to assess the impact of adherence to highly active anti-retroviral therapy (HAART) on long-term immune-virological response in HIV-infected individuals, it was observed that as treatment duration progressed, the probability of non-adherence increased.^28^ Thus, for optimal anti-retroviral outcomes and enhanced viral load suppression, it is vital to ensure that adherence rate of medication is maintained at a higher level at all times for individuals on ART.

Therefore, health authorities need to consider these factors when designing interventions to improve the rate of viral suppression among patients in the study area. It is also vital that more of such studies are replicated in other parts of the country to identify risk factors for virological failure among patients on ART.

There are some limitations to the findings of this study. The first one relates to the nature of the secondary data we used, which permitted us to review only records available in the patients' folders. This did not allow us to do a comprehensive assessment of potential factors associated with virological failure. In addition, we included only patients whose viral loads had been estimated. This might result in underestimation of the true proportion of patients on ART with an unsuppressed viral load. Finally, due to the small sample size drawn from a specific geographical location in Ghana, the findings of the study might have limited generalizability. Notwithstanding these limitations, the study has demonstrated the significant role of socio-economic and patients clinical profile and characteristics as drivers in improving care and attaining the 90-90-90 and the 95-95-95 targets.

## Conclusions

Suppression of HIV viral replication is of great importance in the management of patients living with HIV. This study has identified gender, educational level, comorbidity status, and duration on ART as independent predictors of viral non-suppression among adults living with HIV/AIDS on active ART in Kumasi in the Ashanti Region of Ghana.
